# Designing a test battery for real-world visual search

**DOI:** 10.1038/s41598-025-23111-x

**Published:** 2025-11-11

**Authors:** Charli Sherman, Alasdair D. F. Clarke, Anna E. Hughes

**Affiliations:** Department of Psychology, University of Essex, Colchester, CO4 3SQ UK

**Keywords:** Real-world visual search, Task battery, Human behaviour, Object vision

## Abstract

Although much research has investigated the mechanisms of visual search behaviour in laboratory-based computer tasks, there has been relatively little work on whether these results generalise to more naturalistic search tasks. In addition, work on real-world search has often been carried out by researchers working in very different disciplines, leading to a high degree of task fragmentation. Here, we detail our development of a ‘naturalistic search task battery’, which aims to provide a suite of open-source, reproducible, and standardised real-world search tasks, thus enabling the generation of comparable data across multiple studies and aiding theory and modelling in this area. We show that we can replicate findings from previous search tasks using Lego, demonstrating set-size effects and differences between search conditions. We also show effects of scene regularity using a ‘bookcase’ task where participants search for specific books on shelves. Finally, we use a ‘jigsaw’ task and a ‘Lego building’ task as examples of more complex real-world search tasks and show how we can use them to consider the effects of strategies. Finally, we consider whether performance on one task can predict performance on another, and thus to what extent similar cognitive capabilities underlie diverse ‘real-world’ search tasks.

**Supplementary Information:**

The online version contains supplementary material available at 10.1038/s41598-025-23111-x.

## Introduction

Visual search is a task where participants are asked to find a target among distractors. Much of the work investigating the cognitive processes behind visual search has involved laboratory experiments, carried out on computer screens, and the field has been successful in developing and refining psychological theories of these processes, such as Feature Integration Theory^1^ and Guided Search Theory^2^. The broader class of tasks where participants search for multiple targets (known as ‘visual foraging’) have also inspired theoretical development, such as the Theory of Visual Attention^3^, and the Marginal Value Theorem^4^.

An important factor underlying this success has been the use of standardised experimental paradigms and a relatively small number of outcome measures of interest. This has meant that results are relatively easy to compare across different research groups, aiding theoretical development. While distractors are present in any visual environment or search array, many visual search studies have used search for a target stimulus among discrete distractors, allowing control over the type, similarity, complexity, number and so on. Typical examples would involve a participant searching for such red square amongst green triangles, or a 2 among 5s, and the body of work in this area has enabled general rules about visual search to be developed, such as the fact that there is often a linear relationship between reaction time (for detection, identification and localisation) on these tasks and the number of items in the display^5,6^, and that the slope of the relationship between target-absent and target-present slopes is slightly greater than 2:1^7^. Similarly, visual foraging tasks have shown that when foraging for two classes of stimuli, people switch frequently between target categories when the individual items ‘pop-out’ in what is often termed ‘feature search’ (generating short ‘runs’) but tend to exhaustively search for one target type and then move to the other one (generating long ‘runs’) when focused attention is required to identify the targets via ‘conjunction search’^8^.

In many research projects, the importance of studying visual search has been linked to its real-world applications: for example, understanding search may help understand how people find misplaced keys, or how they search for hazards when driving. Following on from these ideas, there have been an increasing number of studies that attempt to increase the ecological validity of visual search experiments. In some cases, this has involved trying to use more realistic stimuli, though still in the context of a computer-based search task: for example, using continuously textured backgrounds that mimic the power spectra of natural scenes^9^, or asking participants to search for specific objects in photographs of real-world scenes^10^. By increasing the realism of the tasks, researchers have been able to probe aspects of visual search that would not be possible with arbitrary, artificial stimuli, such as scene guidance, where observers use their knowledge of a scene to guide their attention towards areas that are likely to contain the target (e.g. if you need to search for the microwave in an image of a kitchen, you are more likely to search the counters than the floor^11^). However, it is still arguable that most of this work does not really replicate true ‘real-world’ scenarios. While there are examples of tasks that people carry out in their day-to-day life which involve searching on a computer screen (such as radiologists searching for tumours on X-rays, or airport screening^12,13^), many of the search tasks we carry out in real life include factors such as head and body movement, larger-scale search environments, or competing cognitive demands that may be less easy to study using a computer-based task.

When considering non computer-based real-world search tasks, researchers have taken several different approaches. In some cases, studies have aimed to replicate findings from ‘classical’ visual search experiments in physical space, such as replicating linear search slope results by asking participants to search for marbles under film canisters^14^, or by demonstrating foraging-style run-length behaviour in a bead sorting task^15^. Some recent research has used virtual reality to generate naturalistic environments, with examples that include studying inhibition of return in visual search^16^ or foraging tasks^17,18^. Others are more focused on applied questions, such as how operators search CCTV screens^19^, or how people search for food in a supermarket^20^.

One broad question that remains largely unanswered is whether the computer-based laboratory experiments can be taken as a proxy for more ‘real-world’ scenarios, and to what extent visual search can be thought of as being a generalisable process: do the same cognitive processes underlie different versions of searching, or should they all be considered separate tasks? There is good evidence that in some cases, visual search ability does generalise^21^. For example, performance on a high prevalence target search task can predict performance on a low prevalence target task^22^, and visual search assessments have been shown to be good predictors of competence for airport screening^23^. However, there is also recent work suggesting that results from ‘traditional’ visual attentional tasks may not generalise to more realistic scenarios^24^. Clearly, for applied questions, generalisability may not be necessary, or even desirable, as the questions asked by researchers may be highly specific to a particular context. However, we might hope as psychology researchers that there are at least some underlying, predictable factors that help us to explain behaviour (although even within fairly similar laboratory tasks, there is some evidence that performance in one does not predict performance in another^25^). The challenge for real-world visual search is that to date, there has been relatively little overlap in the methods used in different studies, making it difficult to make meaningful comparisons and hampering efforts to create widely applicable, quantitative theories of search behaviour. This has also been identified as a key issue in other areas of psychology (e.g. motor learning^26^) and it has been argued that it is more generally important to adopt a ‘distributed collaborative network‘ approach, designing a range of standard paradigms to address questions of interest within psychology across multiple research groups^27^.

There are a number of real-world visual search protocols that have already been developed that could potentially be built upon or extended. For example, a ‘table top search’ where participants are asked to find a particular item out of a wide range of objects has been used previously to answer questions about the influence of memory for distractor items^28^. A standardised version of this task, involving finding specific coins out of a range of different denominations, and specific colours of CD cases, has also recently been developed for assessing children’s visual search abilities^29^. Similarly, Sauter and colleagues developed a paradigm which used LEGO targets to test a range of classic visual search hypotheses, including set size and feature/conjunction effects^30^, and recent work has also used similar methods to study the effects of active compared to passive search^31^. Others have used a ‘mail box search’ where participants walk into a mailroom containing many mailboxes, and are asked to identify a specific target mailbox^32^.

These types of tasks have a number of benefits: they allow a level of control over the visual environment, while also allowing for a more realistic searching experience than can be achieved in a computer-based task, forming a ‘bridge’ between tightly controlled laboratory studies and uncontrolled real-world interactions. In addition, they allow consideration of aspects of search that cannot easily be studied in a computer-based set up, such as the use of a wider field of view and allowing active search, with participants moving their head and body. Importantly, they all have the potential to be relatively inexpensive and easy to run, making them accessible methods for laboratories without large amounts of funding.

In the current manuscript, we describe an initial attempt to create a ‘real-world visual search’ battery of tasks that we think could serve as a starting point to create methodological coherence in the field. We think that a battery of tasks is helpful as not all search tasks will tap into exactly the same processes (e.g. some, such as table top search tasks require a relatively narrow field of view and little head movement, whereas others such as mailbox-type tasks, involve more movement). In addition, some tasks may be more suited to answering certain questions than others, and as described above, there are inherently interesting scientific questions relating to how well performance on one task can predict performance on another, and what this can tell us about the cognitive processes underlying behaviour. Our tasks range from relatively straightforward tasks that largely replicate experimental tasks carried out in the laboratory to more complex tasks that more closely mimic real-world situations and incorporate a wider range of cognitive processes: for example, when completing a jigsaw, part of the task involves searching for the next piece you are trying to find, but participants also need to plan (‘where to begin this task?’) and incorporate haptic feedback (‘do these pieces fit together?’)

We describe preliminary experiments using readily available, inexpensive and low-tech materials (LEGO, bookcases and jigsaws) with full details of our methods to aid the reproduction and extension of our experiments. We focus specifically on making the protocols as easy to replicate for as wide a range of researchers as possible: for example, the cost of LEGO may be prohibitive for some laboratories, and previous experiments have used relatively large amounts (e.g. 10 boxes of 1500 pieces^30^). Similarly, VR approaches, while extremely powerful, may be out-of-reach for some labs due to the costs and technical expertise required to set up experiments using them. First, we attempt to design ways of replicating the key experimental results from^30^ using smaller stimulus sets, detailed in our *‘LEGO search’* and *‘LEGO dots’* tasks. We also test hypotheses relating to target absent/present search times using a *‘bookshelves’* task. Finally, the *‘LEGO building’* and *‘jigsaws’* tasks are more exploratory, but we aim to show some of the range of hypotheses that could be explored using these paradigms, using both behavioural and head/eye movement data collected using mobile eye tracking devices.

## Methods

### Participants

Our sample included 71 participants (54 females, 17 males). All participants were recruited from the University’s online sampling pool, and the participants had normal or corrected-to-normal vision. The protocol was granted ethical approval by the University of Essex Science and Health subcommittee (ETH2021-0955 and ETH2324-0132). All experiments were performed in accordance with relevant guidelines and regulations. All participants gave informed consent. Note that not all participants were included in the analysed data for each task (i.e. due to eye-movement recording data quality, or because the participants did not finish the full test battery within the hour), so the totals for each task are recorded in the ’data exclusion’ subsection below.

### Equipment

During the task, participants’ eyes were tracked using a Pupil Labs Pupil Core 200Hz Mobile Eyetracker (Pupil Labs, Berlin, Germany). Participants were calibrated and validated before each task using the pupil calibration marker provided by Pupil Labs, to ensure that the field of view for each task was appropriately covered.

### Procedure

Participants first gave consent to take part in the experiment, and initial eye tracking set up was carried out. The order that participants completed the tasks is outlined in Table 1. We swapped the order of the *‘LEGO building’* task and the *‘jigsaws’* task for participants 30-51 (compared to participants 1-29) because we found that some participants took a long time during the *‘LEGO building’* task, so swapping it to be the final task ensured that they completed all the other tasks (even if they did not finish the *‘LEGO building’* task). The *‘bookshelves’* task was the only task carried out in a different location, so this was completed first.

**Table 1:** Table detailing the task order for each subgroup of participants. The *participants* column details the participant numbers in each subgroup. The *order* column details the order of tasks for this group of participants. The *demographics* column details the demographics of each subset.

Participants	Order	Demographics
1–29	Bookshelves, LEGO search, LEGO dots, LEGO building, jigsaws	6 male, 23 female
30–51	Bookshelves, LEGO search, LEGO dots, jigsaws, LEGO building	7 male, 15 female
52–71	LEGO search, LEGO dots, jigsaws	4 male, 16 female

### Experiment 1: hypothesis-driven tasks

We designed three hypothesis-driven tasks. The first two (1A and 1B, *‘LEGO search’* and *‘LEGO dots’*) were designed to be replications of previous work. The *‘LEGO search’* task focused on the differences between feature and conjunction search. Sauter and colleagues found that colour feature search was fastest, followed by the conjunction search, and then shape feature search^30^. The *‘LEGO dots’* search was designed to investigate the set size effect. The previous study found a linear relationship between set size and response time to complete the task^30^. Our tasks therefore aim to replicate these findings at a smaller scale.

In the *‘bookshelves’* (1C) task we explored how participants search for books on a bookcase. While not a direct replication, we aimed to use this task to explore two well known effects in the visual search literature: search for a target within homogeneous distractors is easier than search for a target within heterogeneous distractors^33^, and target-absent searches are slower than target-present searches^7,34^. For this experiment, we therefore had two levels of shelf organisation (organised - representing a homogeneous search; and unorganised - representing a heterogeneous search), in addition to target-present and target-absent trials.

**Fig. 1:**
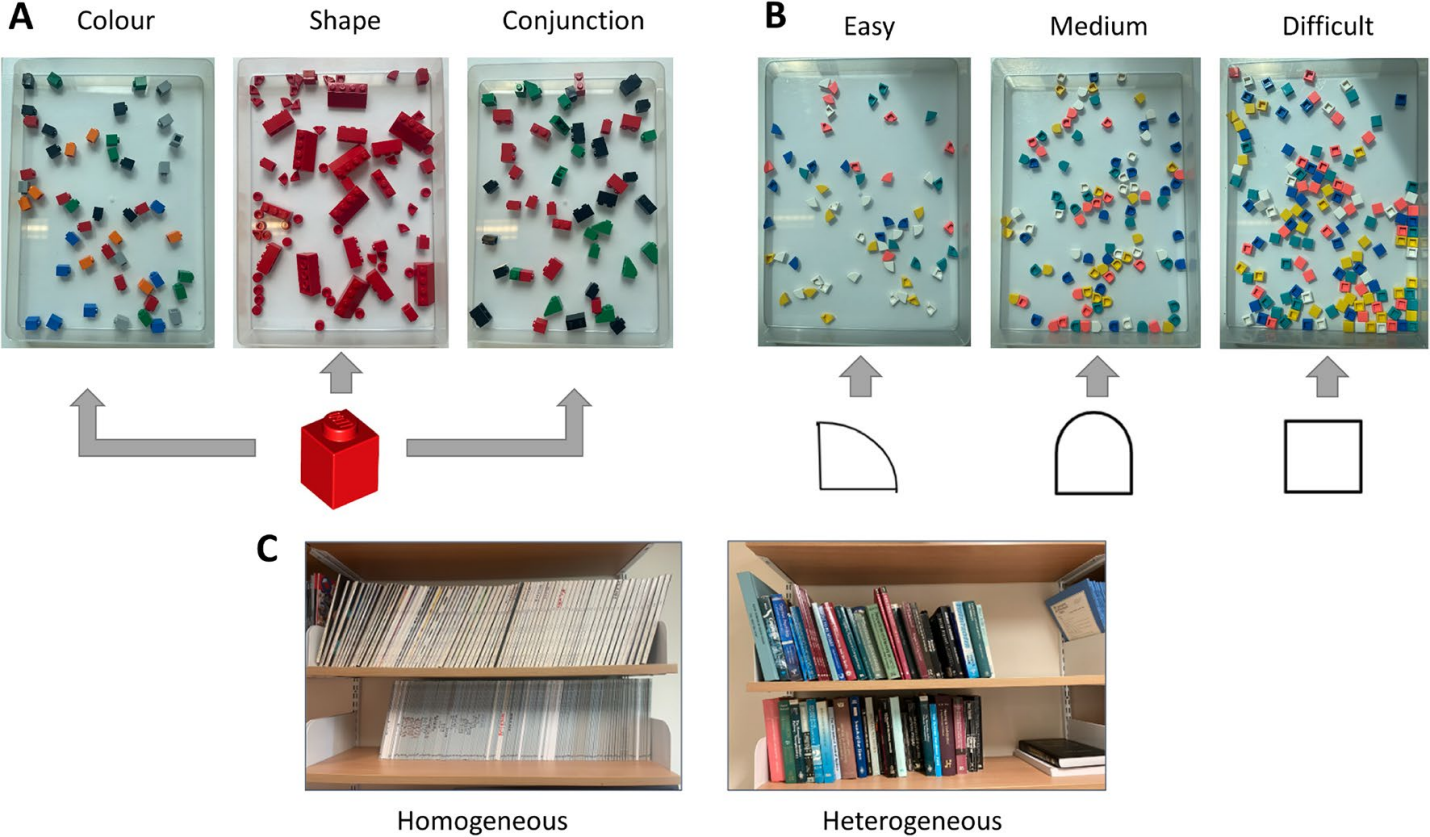
Stimuli and set-up for tasks A B and C.

#### 1A: LEGO search

The participants were presented with two 22 X 16 X 2cm transparent trays. The right-hand tray included the LEGO pieces (from model 10717, 1500 pieces) for the trial, whilst the left-hand tray was empty. This task included three right-hand tray variations to explore feature (*colour* or *shape*) and conjunction searches (*colour and shape*). All trays contained the target pieces (8 x red singular pieces) and the relevant distractor pieces for the specific condition (see Fig. 1, top left). The *colour* based feature search trays included 40 colour variations of singular pieces (8 x light grey, 8 x orange, 8 x blue, 8 x black and 8 x green). *Shape* based feature search trays included 40 red distractor pieces (8 x circle shaped LEGO dots, 8 x corner shaped LEGO dots, 8 x red slope, 8 x red 3-piece, 8 x red 2-piece). The *conjunction* searches included 40 distractors (8 x red 2-piece, 8 x green slope, 8 x green 1-piece, 8 black 1-piece, 8 x red 3-piece; these distractors were all chosen as they had either a similar shape or colour to the targets). The presentation order of the trays were counterbalanced and the trays were shuffled by hand between trials.

The participants sat at an empty table for the initial calibration. Following this, the participants were presented with a printed image of the target piece. The participants focused on a fixation cross on the wall above the table before the trial could begin. The experimenter began the recording and instructed the participant that when the trial began that they were to move all eight target pieces from the search tray on the right to the empty tray on the left. The experimenter then placed the trays on the table in front of the participant and indicated that they could start. This procedure was repeated for the remaining two trials. The participants were presented with one tray at a time and all the subsequent search trays were hidden.

#### 1B: LEGO dots

The basic experimental set up was similar to the *‘LEGO search’* task above. Using one box of LEGO Dots (model 41935, 1040 pieces), three different set size right-hand trays were developed (easy, medium, and difficult). All trays were created in three shape variations (squares, corners, and Norman window). All trays included 20 white target pieces in the trial appropriate shape. The *easy* trays also included 10 X 4 coloured distractor pieces, the *medium* trays included 20 x 4 coloured distractor pieces and the *difficult* trays included 30 x 4 coloured distractor pieces (see Figure 1, top right). All trays were shuffled by hand between participants for randomisation, and the presentation order was counterbalanced between the participants.

The participants sat at an empty table for the initial calibration. Following this, the participants were presented with a printed image of the target piece. The participants focused on a fixation cross on the wall above the table before the trial could begin. The experimenter began the recording and instructed the participant that when the trial began that they were to move all eight target pieces from the search tray on the right to the empty tray on the left. The experimenter then placed the trays on the table in front of the participant and indicated that they could start. This procedure was repeated for the remaining two trials. The participants were presented with one tray at a time and all the subsequent search trays were hidden.

#### 1C: bookshelves

Two wall mounted bookcases were used for the experiment, which each had two shelves of books. One bookcase formed the organised condition. In this condition, the shelves contained numerous volumes of academic journals which had almost identical spines (see Fig. 1, bottom). The second bookcase formed the unorganised condition, which contained books in a variety of sizes and colours. The target books and order of the trials were counterbalanced between all of the participants and the bookcases remained covered prior to the trial to avoid participants familiarising themselves with the book’s location before the trial began.

The participants stood in a fixed position whilst facing away from the covered bookcases and were directed to focus on the wall mounted fixation cross prior to the trial beginning. The researcher uncovered the bookcase and began the recording before showing the participant the target book (away from the bookcase). The researcher then either placed (target-present) or did not place (target-absent) the book in the bookcase. The participants were then instructed to turn around, search for the book, and notify the researcher when they had found the book. The beginning of the trial was taken as when the participant turned around. This procedure was repeated again on the first bookcase before moving onto the second. Participants took part in both a target-present and target-absent trial for both bookcases (unorganised and organised).

### Experiment 2: exploratory tasks

Experiment 2 used an exploratory approach with two tasks that diverge from more traditional visual search paradigms: a *‘jigsaws’* (2A) task where participants put together small puzzles, both with and without the finished image available, to study how having these templates affects search behaviour, and a *‘LEGO building‘* (2B) task where people attempt to follow instructions to build a small LEGO figurine (in this case, an elephant). The latter task somewhat resembles ‘copying’ tasks where participants are given a model, and must recreate it by searching for the relevant elements (among distractors) and moving them into the appropriate configuration^35,36^. Both of these tasks allow us to study broader aspects of search behaviour, such as strategies used (e.g. do people search for particular jigsaw pieces first?) and how visual search relates to other behavioural aspects of complex tasks, such as memory and planning.

#### 2A: jigsaws

The stimuli for the task were a box of Ravensburger Gigantosaurus® aged 3+ puzzles, which included 12, 16, 20 and 24-piece puzzles with a completed size of 19 x 14cm. The jigsaw puzzles were selected to have clear edges of the images and to allow a progression of difficulty, without taking excessive amounts of time to complete. For half of the trials, the participants did not have the puzzle box with the completed picture. The participants completed all four jigsaw puzzles and the ordering of box-present/ box-absent was counterbalanced, with the box-absent trials being presented first for all participants.

The participants fixated on the fixation cross ahead of them while the researcher explained the task. The participants were told that the task involved completing a series of small puzzles. After this the researcher placed the puzzle pieces and the box (in box-present trials) on the table in front of them and the recording was started. Participants repeated these steps over the four trials.

#### 2B: LEGO building

Two transparent 22 X 16 X 2 cm trays of LEGO pieces were used for this task. One tray contained the pre-packaged purple colour set and the other had the pink colour set with twelve white eyes from the LEGO® 10717 Classic box. These provided the participants with all the essential pieces to create a pair of elephants, whilst including additional pieces that meant that participants needed to search for the desired pieces. Participants were provided with double sided print outs of the instructions for the task.

Before the task began, the participants focused on the fixation cross ahead of them whilst the researcher read out the task instructions. Participants were instructed to make the pair of elephants as close to the likeness of those seen on the instructions as possible. The researcher then placed both trays of pieces and the printed instructions ahead of them. The recording then began, and the participants started the task. The recording was stopped upon completion of the task.

### Software

All data processing and visualisation was carried out in R v4.2.2^37^ with the tidyverse (v1.3.2) collection of packages^38^. Bayesian models were fit with brms v2.18.1^39^ and rStan v2.26.13^40^ with tidybayes v3.0.2^41^. See Supplementary Materials or our Github repository for all scripts used for analysis.

### Planned analysis

All response times used in analyses reflect the point from when the participant was told to begin the task to when they completed the task. They were generated by manual checking of the video recordings taken during the tasks.

The data from the tasks in Experiment 1 were analysed using Bayesian multi-level linear models:

$$\begin{aligned} \log (rt) \sim X + (1|Z) \end{aligned}$$

where *Z* is participant ID and *X* encodes the experimental condition. As reaction times typically have a skew, and by definition must be greater than zero, we log-transformed them before fitting the model.

For Task 1A, *X* was a categorical variable encoding whether we were in the colour, shape or conjunction condition. In Task 1B, *X* denoted the number of dots in the search task, defined as $n_{dots}/100$. In Task 1C, *X* included an interaction term, with organisation as one factor (organised vs. unorganised) and target presence as the other factor (present vs. absent). Random slopes were not fit as we only had one trial per participant $\times$ condition.

In Experiment 2, all analyses were post-hoc and are therefore detailed in the relevant Results sections.

### Sample size justification

For Task 1A, previous results^30^ suggested that we could expect an approximately 20% increase in reaction time between the colour and conjunction conditions, which were the most similar in their experiment. Our simulated data suggested that 10 participants would be enough to reliably detect this difference. Similarly, for Task 1B, previous results^30^ suggested that we should expect a 0.18s increase in reaction time per dot, and our data simulations showed that 10 participants should be sufficient to reliably detect this slope. Our experiments should therefore be well powered to detect effects similar to those seen previously^30^ (see code available in the Supplementary Materials for full details of simulations).

Task 1C was not a direct replication, so there was no easy way to predict the effects we might expect. However, our simulations suggest that 40 participants would be enough to detect a reliable difference between organised and unorganised trials if we assumed a mean reaction time for the organised conditions of 1s and 1.2s (for present and absent trials respectively) and a mean reaction time of 1.2s and 1.4s for the unorganised conditions (again, for present and absent trials respectively). We created data for each simulated participant by taking a draw from a normal distribution with the above mean for each trial type, and a standard deviation of 0.5. Further details are available in the code, provided in the Supplementary Materials.

Experiment 2 did not have planned hypotheses, and therefore sample size justifications were not carried out.

### Data inclusion

Full details of data inclusion for each task can be found in Table 2. Note that all participants completed Task 1A, but data from 19 participants were used in the analysis due to an initial error in setting up the tray conditions, leading to one condition having too many distractors. Full data from the excluded participants are available in the Supplementary Materials: we include only the participants who completed the correct version of the task in our main analyses detailed here, but the basic findings replicate when using the excluded participants.

**Table 2:** Table overviewing the data used in each task in this paper. The *participants* column details how many participants were included in each analysis. The *notes* column gives further details about why data were excluded.

Task	Participants	Notes
1A	19	Data excluded due to experimenter error—see main text
1B	70	One participant did not finish the task battery, and therefore did not complete this task. 2 participants (1 and 4) are also missing one condition each (difficult and easy respectively) due to technical issues
1C	43	8 participants were given slightly different instructions, and the remaining 20 did not complete this task
2A	69	2 participants did not complete this task. A further 4 participants did not finish one jigsaw, so they only have data from 3 conditions in the dataset
2B	36	20 participants did not complete this task, 13 participants did not finish the task, and 2 participants had a technical error in data collection
2B (P1)	38	We used a slightly different subset of the 51 participants who attempted the task (as some participants who completed the task did not have high enough quality eye-tracking data, whereas some participants who did not complete the task did complete Page 1 and Page 2, and had good quality eye-tracking data, so were included)
2B (P2)	32	As above for Task 2B (P1)

## Results

### 1A: LEGO blocks

**Fig. 2:**
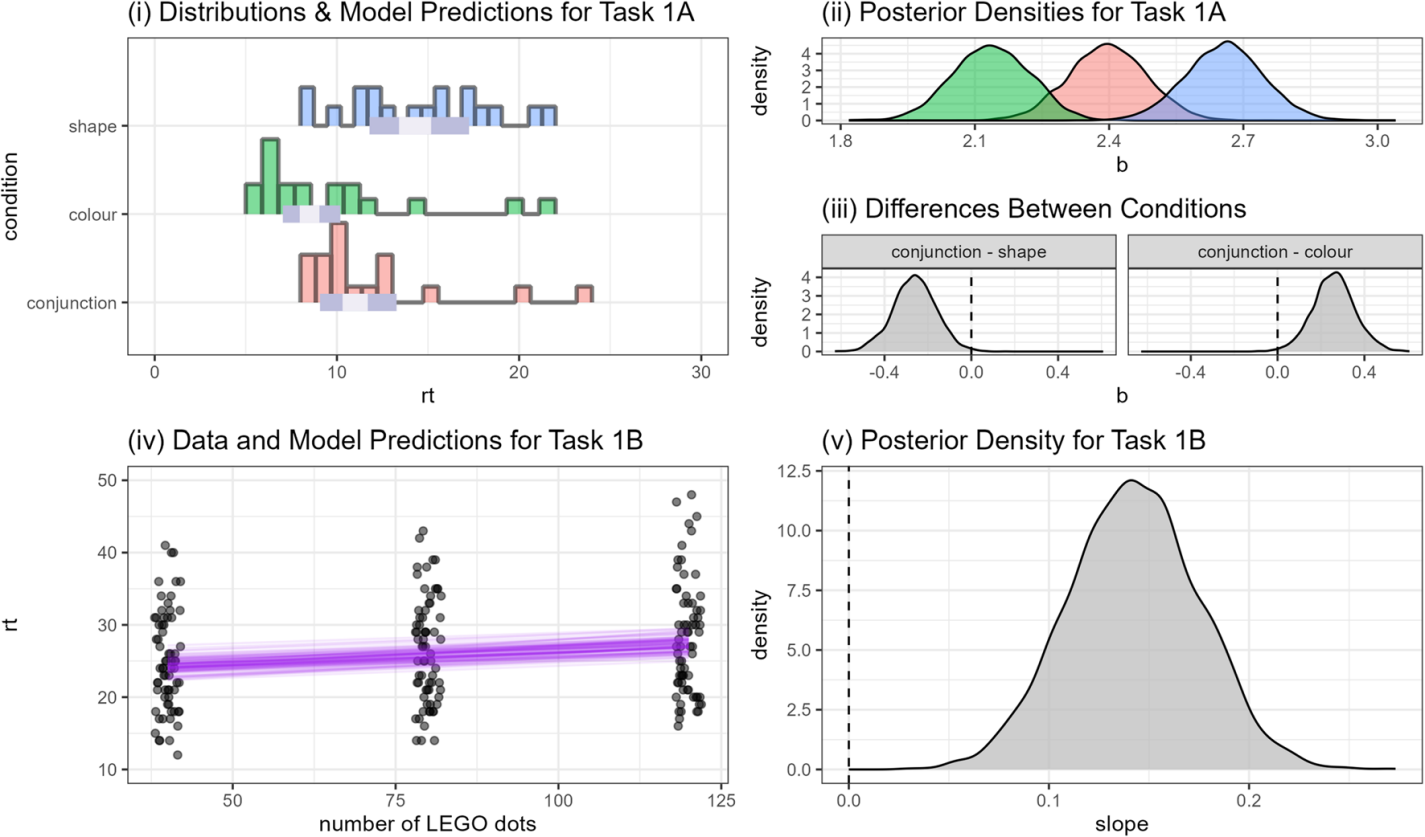
Overview of data and model fits for Tasks 1A and 1B. (i) Histograms show a summary of the empirical reaction times while the intervals indicate the model fits for the mean response. Note: two data points larger than 30 s (85 and 43) are not included in these histograms. (ii) Posterior probability densities for each condition. (iii) Distributions indicating the difference between the two feature conditions and the conjunction condition. (iv) Points indicate empirical RTs while the purple lines show samples from the model’s posterior distribution. Note: two data points above 50 s (93 and 65) are not included in this graph. (v) Posterior probability distribution for the gradient of the search slope.

Results from Task 1A are shown in Fig. 2(i) along with a summary of the Bayesian model (i–ii). As in previous work^30^ we find that the *colour* conditions led to the fastest reaction times, followed by *conjunction* and then *shape*. To investigate this hypothesis in more detail, we first compute the distributions for the difference between the two feature conditions and the conjunction condition (Fig. 2(iii)) and then use these to estimate the probability that the difference between conditions is larger than zero. Doing this results in $Pr(\text {conjunction} - \text {shape}|d) = 0.005$, i.e, given the data there is a 99.5% chance that participants took longer on average in the shape condition compared to the conjunction condition. Similarly, we get $Pr(\text {conjunction} - \text {colour}|d) = 0.995$, which we take as strong evidence that we replicate previous work^30^ in finding that search for colour alone is faster than conjunction.

### 1B: LEGO dots

Task 1B is summarised in Figure 2(iv,v). We can see that as we increase the number of LEGO dots, reaction time increases too. The slope is around 0.14 [CI = 0.08 - 0.21], a relatively weak effect, though the number of distractors is relatively small (for comparison, previous work^30^ used 100–400 distractors, and saw a larger jump in response times for the larger set sizes).

### 1C: bookshelves

**Fig. 3:**
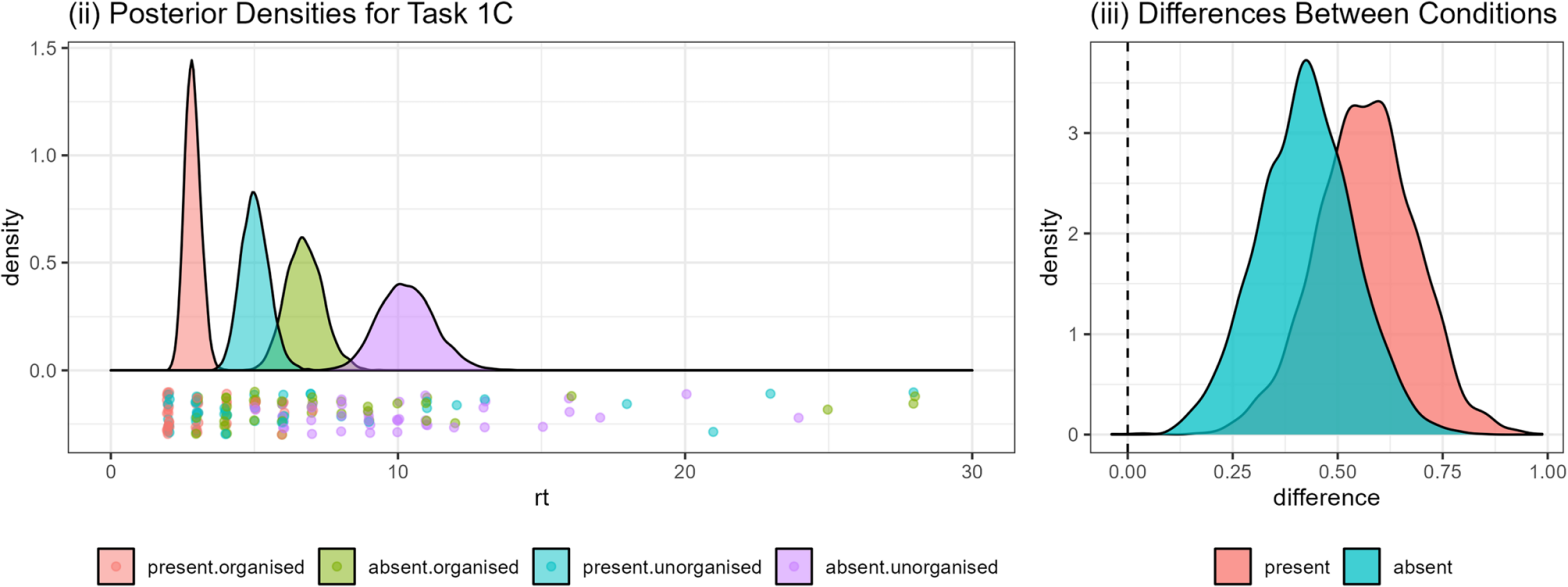
Overview of data and model fits for Task 1C. (i) Points show the response times in each trial, while the density plot shows the estimated posterior probability. Note: 4 data points larger than 30 seconds are not included in this plot. (ii) Distributions indicating the difference between the two conditions for both target absent and present.

The data and statistical model for this task are illustrated in Fig. 3(i). We can see that, as expected, participants took less time to respond in trials in which the target book was present than in those in which it was absent. Furthermore Fig. 3(ii) shows that the manipulation of organisation worked: we can see that participants were faster to search the organised shelf, and this helped for both target present and absent trials: $Pr(\text {unorganised} - \text {organised}|d) = 0.999$.

### 2A: jigsaws

**Fig. 4:**
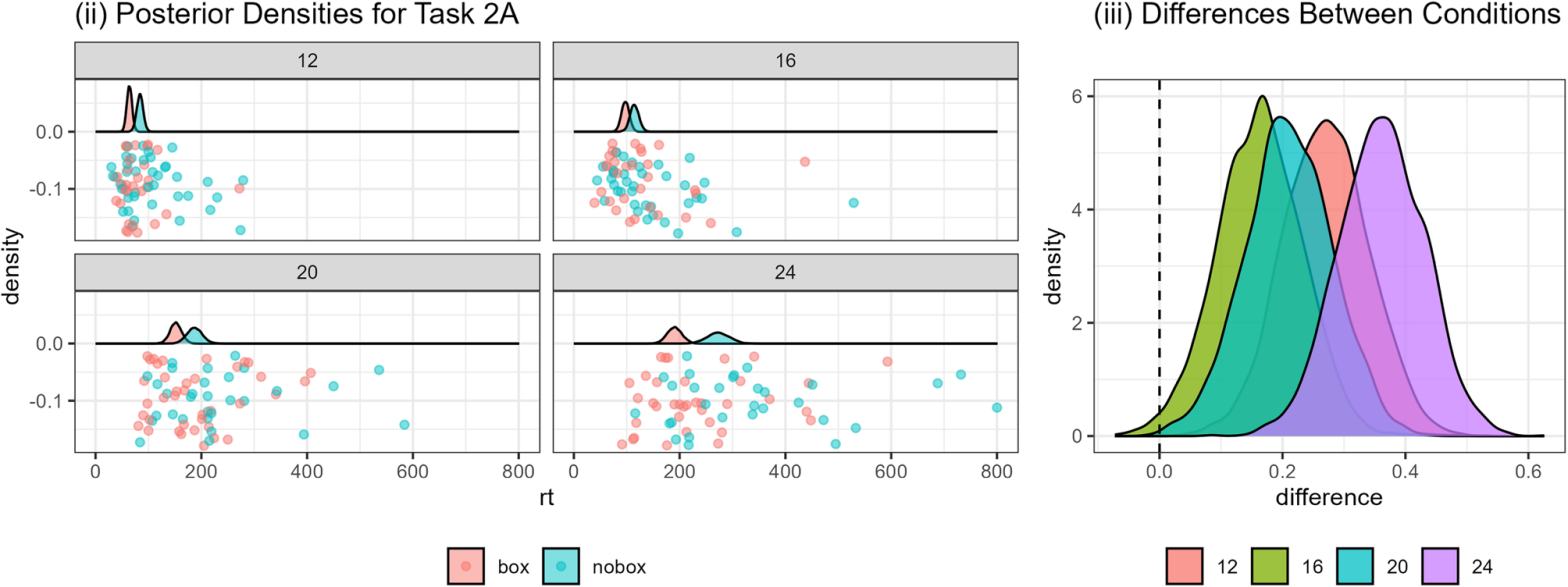
Overview of data and model fits for Task 2A. (i) Points show the response times in each trial, while the density plot shows the estimated posterior probability. Note: 1 data point larger than 800 seconds is not included in this plot. (ii) Distributions indicating the difference between the two conditions for all four jigsaws (i.e. number of pieces).

The data and statistical model for this task are illustrated in Fig. 4(i). We can see that there is an increase in the time taken for each increasing difficulty level of the jigsaws: the jigsaw with 12 pieces is on average quickest to complete, followed by the one with 16 pieces, then 20 and 24 pieces. The variability also increases markedly as the jigsaws get harder, particularly for the ‘no box’ condition. Figure 4(ii) shows that participants were faster to complete the jigsaws when they were able to use the box as a reference: $Pr(\text {nobox} - \text {box}|d) = 0.997$.

**Fig. 5:**
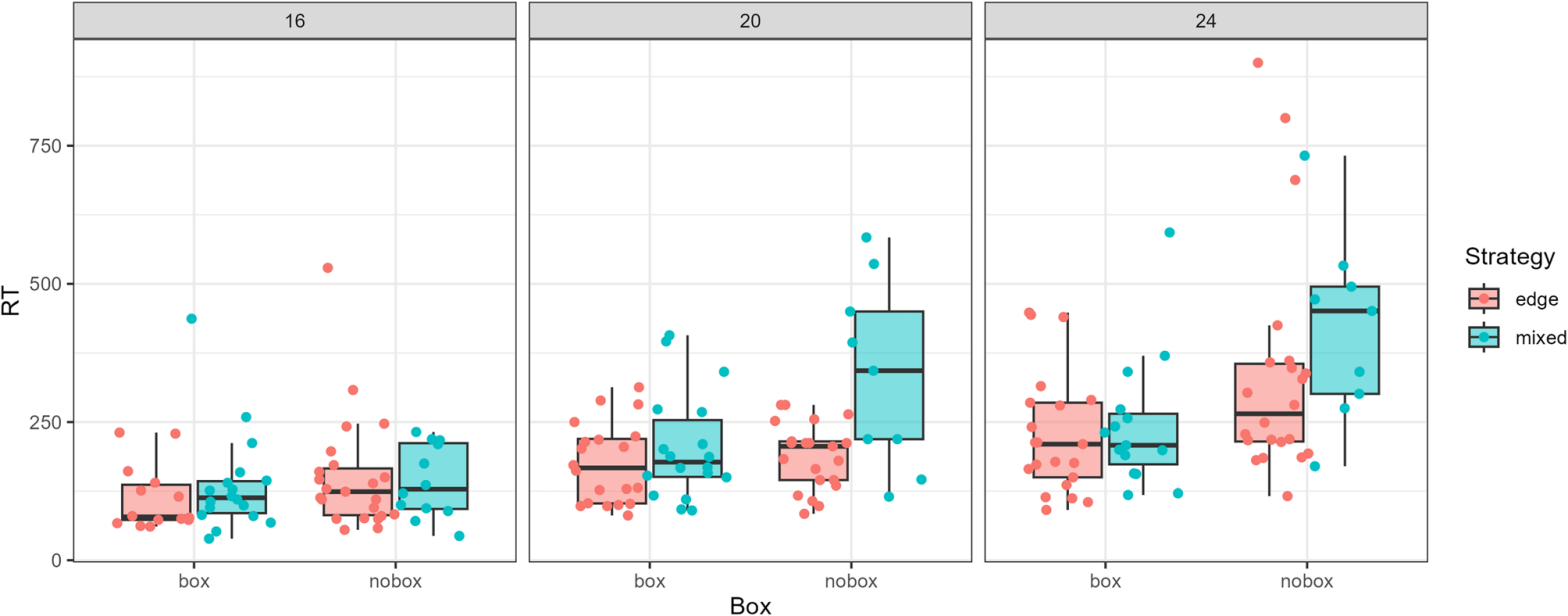
Exploratory analysis of strategy in Task 2A. Here, the first 30 seconds of each video is coded by strategy: each dot then refers to a particular person’s overall reaction time in the task, grouped by strategy, presence of box, and the number of pieces in the jigsaw puzzle.

The video and eye-tracking data provides an incredibly rich set of data for considering questions about strategies in search tasks. As an initial exploratory question, we wanted to ask whether participants’ strategies could be used to predict their performance on the task. We reasoned that for many of the jigsaws, one sensible strategy would be to find the edges/corners first and assemble these before beginning to fill in the centre. For the 12 piece jigsaw, there was in fact only one non-edge piece so we did not use these trials in the analysis. We took the first 30 seconds of each other video and attempted to categorise this section into either an initial ‘edges/corners’ strategy where the participant was clearly trying to build the outer ring of the puzzle, or a ‘mixed’ strategy, where they were also considering central pieces. Figure 5 shows that for relatively easy puzzles (with 16 pieces), strategy does not seem to strongly affect reaction time. However, for more difficult puzzles (with 20 and 24 pieces) without the box, participants who used an ‘edge’ strategy tended to perform better overall on this task. However, interestingly, when participants were able to use the box, both strategies seemed to be similarly effective. Participants were relatively inflexible with their strategies: about 60% used the same strategy throughout all trials they completed (approximately 23% used only a mixed strategy, and 36% used only an ‘edge’ strategy).

### 2B: Lego elephant

Initially, we wanted to characterise where people look during this kind of complex, real-world task. To do this, we manually coded how participants looked at different areas of interest on Page 1 and Page 2 of the LEGO building task. We chose to look at these two pages as they were completed by the majority of participants, and differed in complexity: page 1 involved a couple of relatively simple stages (e.g. step 1 involved finding only a single unique piece), whereas page 2 was more complex, with more pieces to find and a more challenging arrangement of those pieces. We classified eye movements into a number of ‘areas of interest’ for each page: building/hand (where participant were putting LEGO pieces together), instruction 1/3 (depending on which page was being scored), instruction 2/4 (again, depending on which page was being scored), LEGO (where people were looking at the LEGO trays) and turned page (where people flipped the piece of paper that contained the instructions). For Page 2, we also needed to add correction (where people altered something they had previously done), and previous/future instructions (where people looked back to page 1 or ahead to future pages, respectively).

**Fig. 6:**
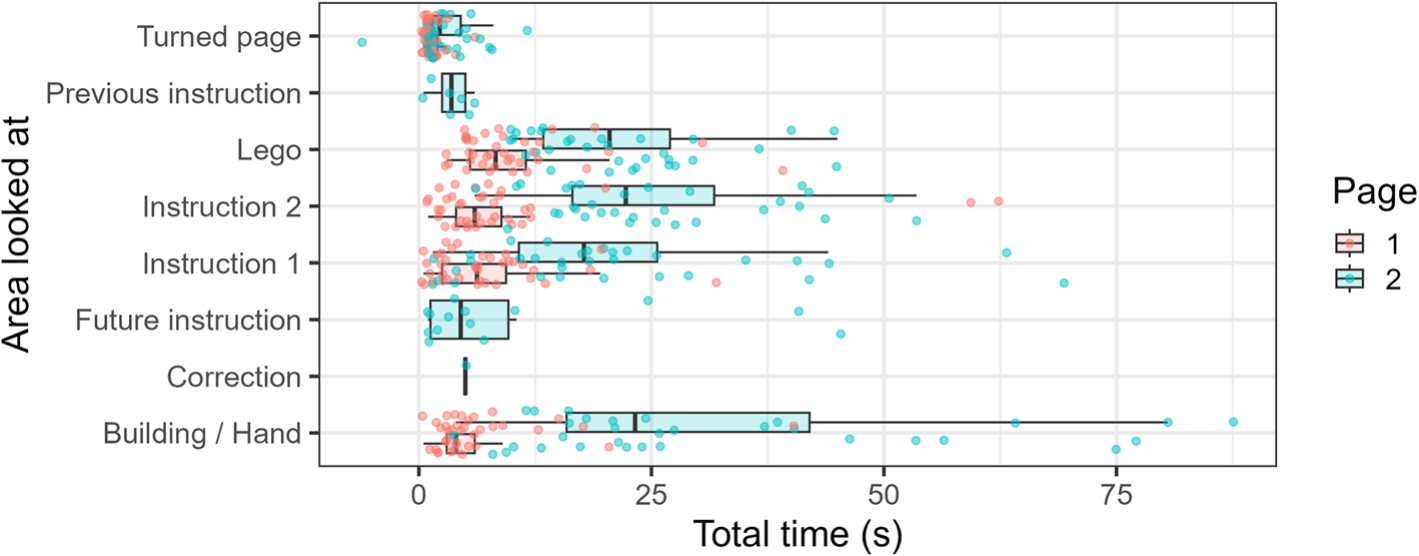
Total time in each ‘area of interest’ per participant for Page 1 and Page 2 of the Lego elephant building task. Each point denotes a different participant.

Perhaps unsurprisingly, Fig. 6 shows that people looked at all ‘areas of interest’ for longer on Page 2: the increased complexity of the task strongly affected all components, including looking at the instructions, searching for the correct Lego pieces and actually building the required structure. It is also worth noting that people clearly plan during this type of task: notably, on Page 2, a number of participants tracked forwards to future instructions (in one case, as far as instruction 13). However, this behaviour was apparent on Page 1 as well. Only 3/38 participants did not look ahead to instruction 2 on Page 1 before beginning to build, despite only needing instruction 1 to be able to complete the initial building sub-task. Indeed, 18 (approximately 37%) of the participants looked at instruction 2 before they even looked at the LEGO. This type of behaviour appears to share some similarities with look-ahead fixations, that have been defined as “fixations on objects not relevant to the immediate sub-task, but relevant for a future sub-task”^42^, and have been observed in a range of behaviours, including walking^43^.

Interestingly, our data also show signs of ‘look back’ behaviour, where people go to check previous instructions: on Page 2, a number of participants went back to look at Page 1, and even on Page 1, the majority of participants (31, approximately 82%) ’tracked back’ to instruction 1 after they had already looked at instruction 2.

**Fig. 7:**
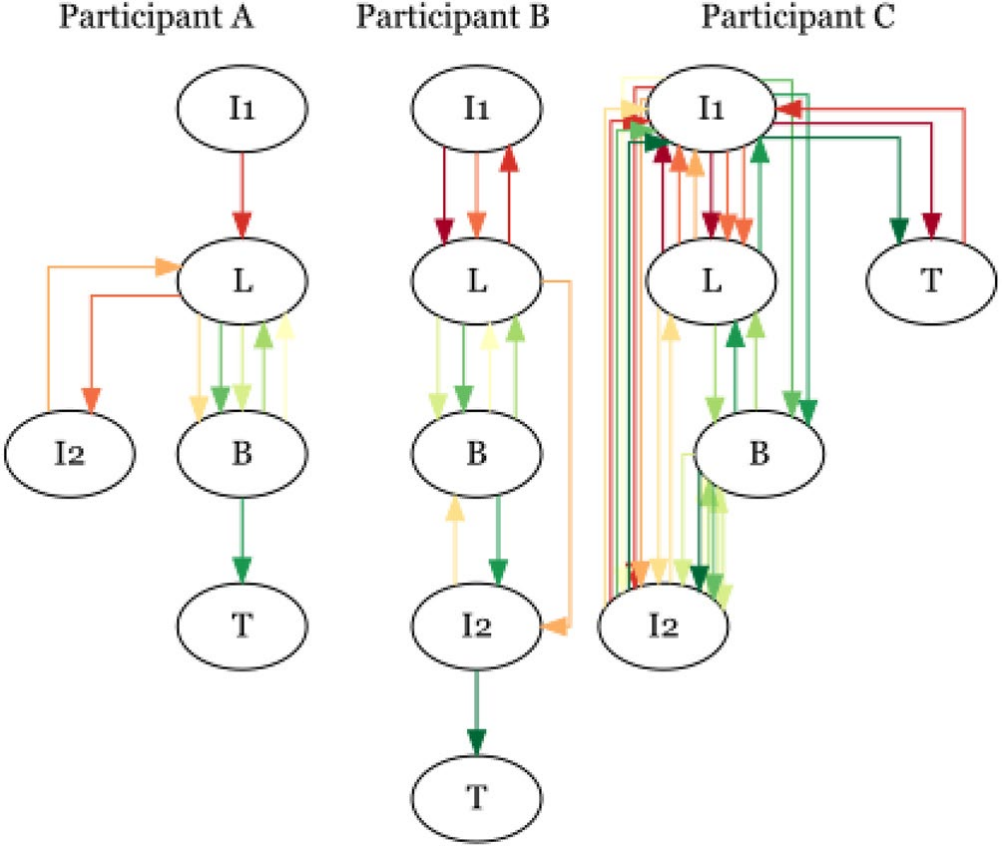
Three example participant gaze strategies, showing the sequence of where they looked on page one of the LEGO building task. I1 = instructions 1, I2 = instructions 2, L = LEGO, B = building, T = turned page. Temporal order is encoded by the colour of the arrow (starting from red and moving through orange/yellow to green). Participant A completed page one in 17s, participant B in 15s and participant C in 39s.

We can also use our data to map out the time span of looking behaviour for individual participants. Figure 7 shows how participants looked at the different areas of interest on Page 1 of the LEGO building task. Some participants (such as Participants A and B in Fig. 7) followed a relatively straightforward pattern, looking at the instructions early in the task, then switching between the LEGO tray and where they were building the elephant, before turning the instructions page over. However, some participants (such as Participant C in Fig. 7) had much more complex strategies, incorporating more ’checking’ behaviour where they returned to the instructions regularly throughout the task.

During the building task, the key ‘visual search’ component is where participants are looking through the LEGO for the pieces they need. If visual search ability is an important predictor of performance in this task, we might expect to see a correlation between visual search time and overall time to complete the whole task. However, for both Page 1 and Page 2, there does not seem to be good evidence for this correlation (Spearman’s rho = 0.24, p = 0.21 for Page 1; Spearman’s rho = 0.26, p = 0.23 for Page 2).

### Overall correlations across tasks

**Fig. 8:**
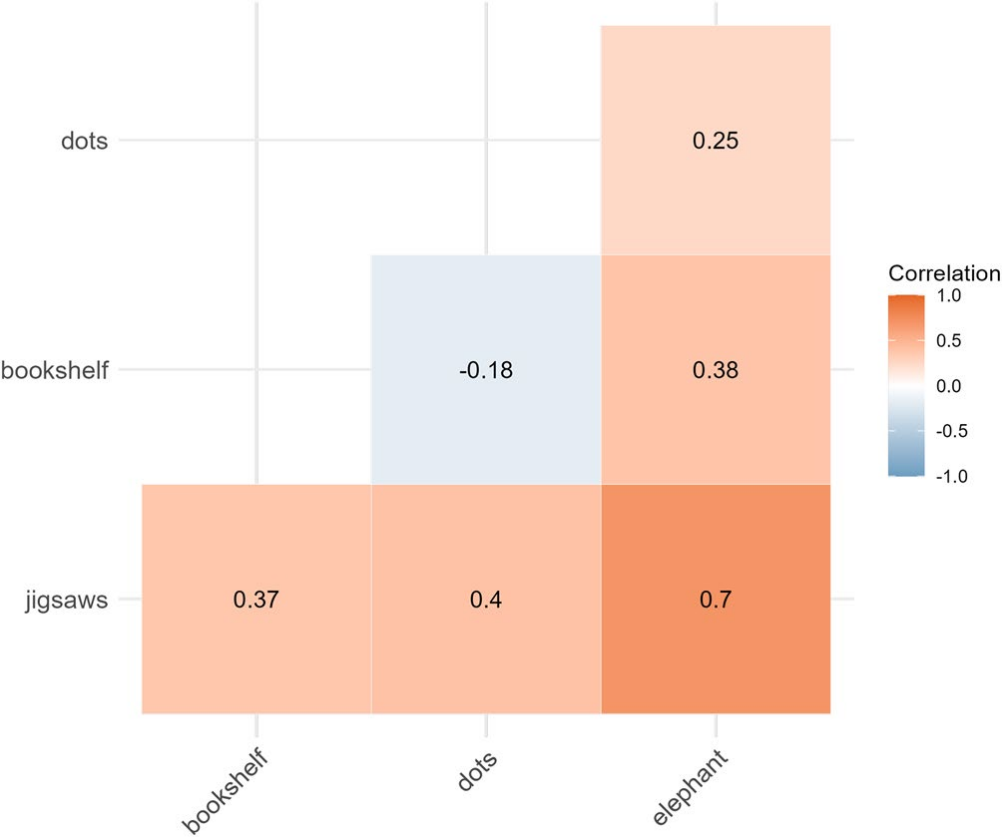
Correlations between the different tasks in our test battery.

All the tasks in the test battery contained some components of ‘visual search’ and therefore we might expect performance on them to correlate. In the 27 participants who had a full dataset (excluding the *‘LEGO dots’* task, as this was run with only a small subset of participants), the correlation coefficients can be seen in Fig. 8. While the small sample size means that these correlations should be considered tentative and exploratory, there were generally positive correlations between tasks. This is particularly interesting given that these tasks are fairly variable, and previous research has shown that performance in relatively similar laboratory-based visual search tasks is not correlated^25^.

## Discussion

The overall aim of this series of experiments was to begin development a ‘real-world visual search’ battery of tasks that could be used more broadly to address questions about visual search behaviour beyond computer-based studies. Our approach made use of simple, easily available and relatively low cost stimuli (LEGO, books on a bookshelf, jigsaws). By recording both video and eye movement behaviour, these tasks give us a rich set of data which we think can be used to answer a wide range of interesting scientific questions.

Our results from Experiment 1 broadly support the key findings from search paradigms carried out on computer screens: for example, the increase in reaction time with increasing set size, the increased difficulty of search in ‘unorganised’ heterogeneous environments compared to ‘organised’ homogeneous environments, and that search is faster when the target is present compared to when it is absent. Our work therefore adds to the increasing body of knowledge that classic visual search effects seem to generalise beyond simple lab tasks^14,16^.

We also replicate previous findings by Sauter and colleagues^30^ in which colour feature search was fastest, followed by conjunction search and then shape feature search. This is interesting because this is not what might be predicted if considering the broader literature on visual search, where conjunction search is generally taken to be slower than feature search. As discussed in their paper, the slow shape feature search may at least partially reflect the fact in an interactive, 3D environment, shape is not a good candidate for a ‘feature’ as originally defined by visual search models^1^, as the targets are viewed in a range of orientations and therefore vary relatively widely in appearance. This effect may be heightened by the fact that the target in our experiment was displayed to participants in a ‘prototypical’ 2D image, athough Sauter and colleagues showed participants real target exemplars in their experiment and found very similar results. Our results therefore point to how we might need to broaden our thinking beyond 2D displays to fully explain visual search findings in the ‘real world’.

We also carried out several more exploratory experiments. In our *‘jigsaws’* paradigm, we saw that people tended to be faster at completing a jigsaw when they were able to use the box as a reference, as might be expected. We also saw some suggestive evidence that people’s strategies on this task might affect how long they took to complete it. In the *‘LEGO elephant’* task, it was clear that participants both planned ahead and also checked back regularly during the task, although there was huge variability in how people approached the building process. One interesting aspect of this paradigm is that we can dissect out the ‘visual search’ component (i.e. when people are searching the LEGO for pieces) and analyse to what extent performance on this task correlates with overall performance on the task. Initial analyses did not find evidence for this link. However, we want to re-iterate that we advise against over-interpreting our analyses, as we did not have pre-specified hypotheses for these experiments. Instead, our results should be considered as pilot findings that are predominantly intended to generate ideas and hypotheses for future work.

Our results also speak to another current debate in the visual search literature: to what extent does performance correlate across different visual search tasks? If we consider ‘visual search’ to be a basic cognitive function, we might expect that for any given individual, their performance across different types of visual search task should be similar. However, recent studies have found no evidence for this^25^. Here, we tentatively suggest that one reason for this might be that the ‘visual search’ aspects of the task are not the key ones in determining variations in performance: we saw little evidence in our LEGO elephant task that the time taken to complete the visual search portions of the task (e.g. searching the LEGO trays for pieces) correlated with overall time to complete the task. However, we did find at least some evidence that overall reaction times across our tasks correlated, suggesting perhaps that some other factor (that is minimised in tightly controlled laboratory conditions) could be considered a more consistent individual difference. For example, our test battery may be picking up on a general ‘spatial manipulation’ ability (as the LEGO elephant and jigsaws task showed the highest correlation with each other). This is of course highly speculative based on the data presented: here, we mostly want to emphasise that thinking about how we could break down tasks into different processes could help to reconcile the folk wisdom that suggests that we might expect to see correlations between performance on ‘cognitive’ tasks with the empirical data to date that have shown no evidence for this^25^.

We have also tried to demonstrate some of the broader questions that could be asked with this test battery, although our analyses are clearly limited, with many more questions and approaches possible. One key limitation with these types of ‘real-world’ data has always been the sheer number of person-hours needed to manually code complex video data: however, there are exciting new developments in computer vision that are beginning to make automatic tagging in videos possible^44,45^, which opens up a wealth of analysis possibilities. For example, if each jigsaw piece can automatically be tagged on each frame of a video, we could look in detail at the sequence of eye movements or piece selections in order to understand in more depth the strategies that people use to complete the task. Similarly, being able to automatically tag the books in a bookshelf could allow understanding of search strategies in this task, such as whether people adopt a ‘scanning’ pattern, or focus on trying to match target features.

In conclusion, we have devised an initial set of tasks for a ‘real-world search’ battery that we think are flexible, relatively inexpensive and hopefully diverse enough to help answer a wide range of questions. We think that taking this approach may help to understand the generalisability of current research on visual search, but may also be particularly exciting in trying to better understand how visual search behaviour interacts with a broader set of cognitive abilities, such as strategy choices, planning, and memory. We hope that others find our ideas interesting, and we would be excited to see them used and developed further by other researchers.

## Supplementary Information

Below is the link to the electronic supplementary material.

Supplementary Information 1.

Supplementary Information 2.

Supplementary Information 3.

Supplementary Information 4.

Supplementary Information 5.

## Data Availability

The datasets generated and analysed during the current study are available in the real world visual search repository, available on Github.
